# High density marker panels, SNPs prioritizing and accuracy of genomic selection

**DOI:** 10.1186/s12863-017-0595-2

**Published:** 2018-01-05

**Authors:** Ling-Yun Chang, Sajjad Toghiani, Ashley Ling, Sammy E. Aggrey, Romdhane Rekaya

**Affiliations:** 1 Department of Animal and Dairy Science, University of Georgia, Athens, GA 30602 USA; 2 Department of Poultry Science, University of Georgia, Athens, GA 30602 USA; 3 Institute of Bioinformatics, University of Georgia, Athens, GA 30602 USA

**Keywords:** SNP prioritizing, Genomic selection, High density

## Abstract

**Background:**

The availability of high-density (HD) marker panels, genome wide variants and sequence data creates an unprecedented opportunity to dissect the genetic basis of complex traits, enhance genomic selection (GS) and identify causal variants of disease. The disproportional increase in the number of parameters in the genetic association model compared to the number of phenotypes has led to further deterioration in statistical power and an increase in co-linearity and false positive rates. At best, HD panels do not significantly improve GS accuracy and, at worst, reduce accuracy. This is true for both regression and variance component approaches. To remedy this situation, some form of single nucleotide polymorphisms (SNP) filtering or external information is needed. Current methods for prioritizing SNP markers (i.e. BayesB, BayesCπ) are sensitive to the increased co-linearity in HD panels which could limit their performance.

**Results:**

In this study, the usefulness of F_ST_, a measure of allele frequency variation among populations, as an external source of information in GS was evaluated. A simulation was carried out for a trait with heritability of 0.4. Data was divided into three subpopulations based on phenotype distribution (bottom 5%, middle 90%, top 5%). Marker data were simulated to mimic a 770 K and 1.5 million SNP marker panel. A ten-chromosome genome with 200 K and 400 K SNPs was simulated. Several scenarios with varying distributions for the quantitative trait loci (QTL) effects were simulated. Using all 200 K markers and no filtering, the accuracy of genomic prediction was 0.77. When marker effects were simulated from a gamma distribution, SNPs pre-selected based on the 99.5, 99.0 and 97.5% quantile of the F_ST_ score distribution resulted in an accuracy of 0.725, 0.797, and 0.853, respectively. Similar results were observed under other simulation scenarios. Clearly, the accuracy obtained using all SNPs can be easily achieved using only 0.5 to 1% of all markers.

**Conclusions:**

These results indicate that SNP filtering using already available external information could increase the accuracy of GS. This is especially important as next-generation sequencing technology becomes more affordable and accessible to human, animal and plant applications.

**Electronic supplementary material:**

The online version of this article (10.1186/s12863-017-0595-2) contains supplementary material, which is available to authorized users.

## Background

Large-scale genotyping for single-nucleotide polymorphisms (SNPs) has provided an unprecedented resource to study associations between traits and genomic variation and to compute genomic enhanced breeding values (GEBVs). Although a detailed dissection at the genetic level of these complex traits is still largely elusive, continuous improvements in the quality and diversity of high-throughput data, as well as the development of more sophisticated statistical and computational tools, are quickly moving us towards a better understanding of the genetic basis of these traits. Genomic selection (GS) is rapidly becoming the tool of choice for genetic evaluation of several livestock species due to an increase in accuracy and substantial reduction of the generation interval [1–5]. Genomic selection is currently being implemented either through a multiple regression (RM) or variance component (VC) based models. The RM approach consists of a multiple step procedure where SNP effects are first estimated in a training population and then validated in a separate data set. Several methods have been developed and used to implement this approach [6–15]. Although these methods have different statistical and biological assumptions regarding the data generating process, they tend to yield similar results in most cases, at least when low- to moderate-density panels are used; differences are largely due to the genetic architecture of the trait, the genetic relationships between individuals in the data, and the chosen prior information.

Next generation sequencing (NGS) has dramatically changed the speed, coverage and costs of sequencing whole genomes. Several sequencing efforts, including the 1000 Bull Genomes Project [16], are underway, while several thousand humans and animals have already been fully sequenced (The [17]). These projects are crucial for characterizing the source of genetic variation. In fact, 84 and 31.8 million common and rare variants have been already identified in human and dairy cattle, respectively (The [17, 18]). Although a majority of these variants are rare (MAF <1%), over 8 million common SNPs (MAF > 5%) have already been identified in humans. Thus, it is already a reality that genome-wide association study (GWAS) and ultimately GS will be implemented using several millions of directly- or indirectly-imputed sequence variant genotypes. GWAS using 17 and 19 million SNPs were carried out in human (The [17]) and dairy cattle [16] applications, respectively. Although theoretically there are no doubts about the potential usefulness of the sequence data in GWAS and GS, major challenges are limiting the harnessing of these benefits.

The major problem of the analysis of high dimensional SNP data and sequence variant genotypes stems mainly from the high dimensionality of the parameter space. When all variants are considered (i.e., BayesA), the highly informative prior will lead to excessive shrinkage that together with the high linkage disequilibrium (LD) precludes the identification of causative mutations or even of significant tag variants. As the effect of a QTL (often small for complex traits) is distributed in a non-trivial manner between all markers that are in LD with the causal mutation, there is little statistical power to accurately estimate its effect. Given these limitations, filtering (prioritization) of variants to be included in the association models has become a necessity. Traditionally, SNP filtering is conducted based on certain statistical criteria such as *p*-values for single-marker analyses [19, 20] or quality of fit and model determination for Bayesian procedures such as BayesB [6] and BayesR [21]. The latter showed some superiority for certain traits in the presence of low- and moderate-density marker panels compared to models that including all markers. However, they still suffer, although to a lesser degree, from high false positives, multiple testing problems, high LD and small SNP effects which have hampered at different degrees the efficiency of these methods. Consequently, with the current density of sequence variants, it is clear that statistical discriminatory criteria alone will not be enough to prioritize influential variants, and enlistment of additional external sources of information seems to be an attractive alternative. BayesRC [22] which is an extension of BayesR through the inclusion of biological prior information (variant type, location in differentially expressed genes), has only led to slight increase in accuracy compared to BayesR [18].

The limited success so far of these SNP/variant-prioritizing methods is due to several reasons: 1) the “artificial” reduction in the number of parameters in the model. Although marker prioritization methods based on statistical criteria (BayesB, and BayesR) reduce the number of parameters (variants) fitted in the association model in every round of the iterative process, the total number of unknowns to be inferred in each round is at least equal to the number of parameters in a fully parameterized model (i.e., BayesA). This is due to the need to identify those markers with zero effects which is often accomplished either through a Metropolis-Hastings step or through the estimation of indicator variables in a data augmentation approach; 2) currently available biological information is often limited (tissue specific, time specific, etc.) and with a high noise-to-signal ratio (gene expression, methylation profiles, etc.); and 3) small QTL effects in LD with a large number of variants.

Consequently, other sources of prior information need to be investigated. Livestock species are under heavy artificial selection. The signature of such selection pressure can be traced through changes in allele frequencies of markers in LD with QTL. F_ST_, a measure of allele frequency variation among sub-populations, provides a tool to reveal selection sweeps [23] and can be used to identify SNPs under selection pressure. In this study, a simulation was carried out under different marker densities and complexity of the genetic model to evaluate the usefulness of F_ST_ scores as an external source of information to prioritize SNP markers in the association models and to compare its performance with currently used approaches.

## Methods

### Simulated SNP genotypes and phenotypes

Simulation was carried out using QMSim software [24]. A historical population was generated based on random mating of 8000 animals for 300 generations followed by an additional 15 generations of random mating with population size ranging between 12,000 and 17,000 animals. This random mating was carried out to initialize LD and to establish mutation-drift equilibrium in the historical population. The founder population or generation zero (G0) was created from the last historical generation based on 1500 males and 15,000 females. The mating of these individuals was random and no selection was considered at this step. After G0, four generations were simulated. The third generation (G3) was used to detect selection signatures and the last one (G4) was used to evaluate the proposed approach.

In the last four generations (G1 to G4), animals were selected base on their estimated breeding values (EBVs). Replacement rate for males and females were set to 50 and 20%, respectively. In all generations, one progeny per dam and a sex ratio of 50% were assumed. Only animals in generation three and four were assumed to be genotyped. In order to mimic high-density marker panels, a 10-chromosome genome was simulated with uniformly-distributed 200 K and 400 K SNP markers, resulting in a density similar to a bovine chip of 600 K and 1 million SNPs, respectively. The additive effects of one hundred QTL were sampled either from a Gamma distribution with shape parameter equal to 0.4 or predefined as a fraction of the total genetic variance. In the predefined scenario, QTL effects were set to explain at least 0.5% of the genetic variance. Both SNP markers and QTL in all simulated scenarios were assumed to be bi-allelic, and no marker loci overlapped with the QTL. A detailed description of the simulated genome structure of the different scenarios is presented in Table 1.

**Table 1:** Descriptive statistics of simulation schemes

Historical Population (HP)	
Number of generation	315
Mutation rate for markers	10^−4^
Mutation rate for QTL	10−4
Founder Population (G0)	
Number of generation	3
Number of male	1500
Number of female	15,000
Selection Population (G3)	
Number of chromosomes	10
Length per chromosome (cM)	100
Number of markers per generation	200,000/400,000
Marker distribution	Evenly spaced
Number of QTL per generation	100
QTL distribution	Randomly distributed
QTL effect	Sampled from gamma with shape 0.4
Heritability	0.4
Genetic variance	0.4
Residual variance	0.6

The phenotype consisted of one trait with 40% heritability. Phenotypic variance was set equal to one and the residual variance was adjusted in each scenario to maintain the heritability constant at 0.4. The true breeding value of an individual was equal to the sum of the QTL additive effects. Phenotypes were generated by adding random errors, sampled from a normal distribution with zero mean and dispersion equal to the residual variance.

### Measure of selection pressure as source of external information

Wright’s F statistics [25] are fixation indexes that measure the rate of fixation through the increase in homozygosity. In particular, F_ST_, a measure of population structure, is one of the most frequently used scores in the field of genetics. It measures the rate of genetic differentiation between subpopulations through the assessment of the changes in allele frequencies. The larger the F_ST_ values, the higher the genetic differentiation [26–29]. Among its multiple uses, F_ST_ can be used to assess signatures of natural and artificial selection.

Although there are several methods to estimate F_ST_ [30–33], the global estimator proposed by Nei [30] was used in this study. Animals in generation G3 were divided into three sub-populations based on their simulated phenotype (below the 5 quantile [**S1**], between 5 and 95 quantiles [**S0**], and above the 95 quantile [**S2**]) and F_ST_ score for a given locus was calculated as:

$$ {\displaystyle \begin{array}{c}{F}_{ST}=\frac{H_T-{H}_S}{H_T}\\ {}\mathrm{with}\ {H}_T=2\ast p\ast q,{H}_S=\frac{H_{S1}\ast {\mathrm{n}}_{S1}+{H}_{S2}\ast {n}_{S2}}{n_{S1}+{n}_{S2}},\mathrm{and}\ {H}_{Si}=2\ast {p}_{si}\ast {q}_{si}\end{array}} $$

where, *p*_*Si*_ and *q*_*Si*_ are the allele frequencies in subpopulation *i*, *n*_*s*1_ and *n*_*s*2_ are the number of individuals of the subpopulations, *H*_*S*_ is the average of sub-population heterozygosities and *H*_*T*_ is the heterozygosity based on the total population.

Animals in subpopulations S1 (below 5 quantile) and S2 (above 95 quantile) of the third generation of simulation data (G3) were used to calculate the F_ST_ scores. A total of 1500 genotyped animals equally divided between both groups were used. Three heuristically defined threshold values of F_ST_ scores (Table 2) were used to select SNPs that are potentially under genetic differentiation. For 200 K SNP panels, the number of selected SNPs was 935, 1956, and 4932 for the three threshold values in the gamma distribution scenario, respectively. The number of selected SNPs was 1076, 2171, and 5620 in the predefined distribution scenario, respectively.

**Table 2:** Preselected SNPs based on different cutoff values for the FST scores and different simulation scenarios

Panel density	QTL effects^1^	Quantile	F_ST_ Score^3^	Selected SNPs^4^
		99.5	0.02	935
	Gamma^5^	99.0	0.01	1956
		97.5	0.004	4932
200 K				
		99.5	0.009	1076
	Predefined^6^	99.0	0.007	2171
		97.5	0.005	5620
		99.5	0.015	2078
	Gamma	99.0	0.009	3586
		97.5	0.004	10,178
400 K				
		99.5	0.009	2036
	Predefined	99.0	0.007	4646
		97.5	0.004	10,651

^1^Distribution used for the simulation of the QTL effects, ^2^quantiles of the F_ST_ score distribution, ^3^cutoff point for the fixation index (F_ST_), ^4^number of selected SNPs based on the F_ST_ score cutoff, ^5^Gamma distribution with shape parameter equal 0.4, and ^6^QTL effects pre-defined to explain at least 0.5% of genetic variance each

### Data analysis

Each simulated data set was analyzed using BayesB, BayesC, and the proposed method where SNPs selected based on their F_ST_ scores are used as explanatory variables in a regression model similar to BayesA. Implementation of BayesB and BayesC was carried out using GenSel software [34] with (1-π) values set equal to 0.9, 0.95, 0.98, or 0.99. Scaled inverted Chi square prior distributions were assumed for the genetic and residual variances with scaling factors equal to the true values used in the simulation and degrees of freedom of 1 and 4, respectively.

The general statistical model used for analysis in BayesB, BayesC and the proposed method can be presented as:

$$ {y}_i=\mu +\sum \limits_{j=1}^p{X}_{ij}{\beta}_j{\gamma}_j+{e}_i $$

where *y*_*i*_ is the phenotype for individual *i*; *μ* is an overall mean; *X*_*ij*_ is the genotype of individual *i* for SNP *j* taking the value of 0, 1, or 2; *β*_*j*_ is the effect of the SNP *j*; and *γ*_*j*_ is an indicator factor that takes the value of 1 if SNP *j* is included in the model and 0 otherwise. For the proposed method, *γ*_*j*_ was equal to 1 for all preselected SNPs. *e*_*i*_ is the error term and *p* was equal to the preselected SNPs for the proposed method or the total number of SNPs times (1- *π*) for BayesB and BayesC.

Point estimates of the SNPs effects were used to compute the estimated genomic breeding values as:

$$ {GEBV}_i=\sum \limits_{j=1}^p{z}_{ij}\widehat{a_j} $$

where $\widehat{a_j}$ is the estimated effect of SNP *j.*

Genomic and phenotype accuracies were calculated based on the correlation between the true breeding values and the GEBVs and between the GEBVs and the observed phenotypes adjusted for the systematic effects.

For each simulated data set, randomly 10,000 genotyped animals in the third generation (G3) were assigned to the training population and randomly 5000 genotyped animals in the last generation (G4) were used for validation. Each simulation scenario was replicated 5 times. For BayesB and BayesC, four (1 − *π*) values (0.99, 0.98, 0.95, and 0.9) were evaluated.

## Results and discussion

### Distribution of QTL and estimated F_ST_ scores

**Fig. 1:**
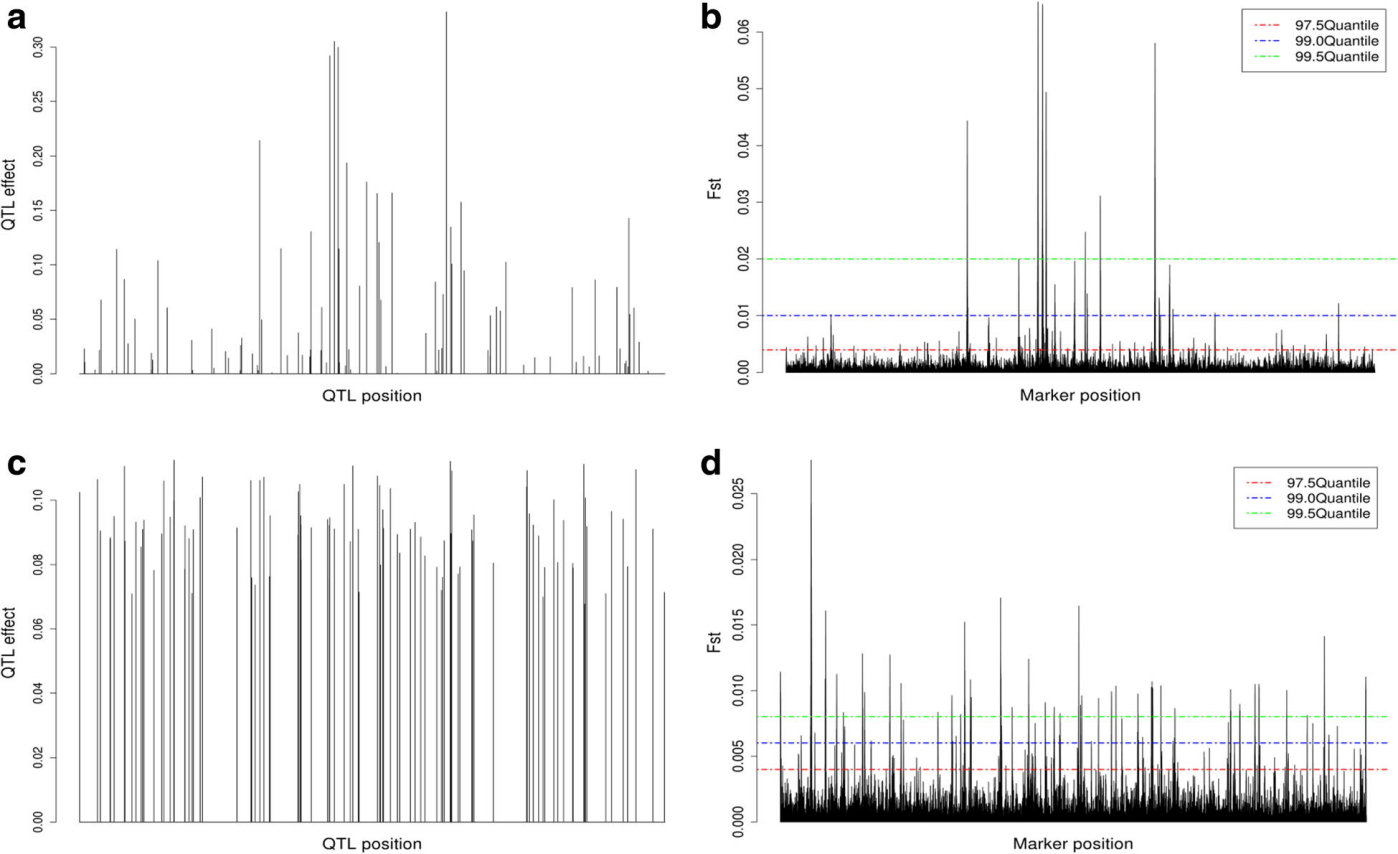
Distribution of the simulated quantitative trait loci (QTL) along the ten chromosomes when their effects were simulated from a gamma distribution (**a**) or predefined (**c**) and their associated F_ST_ scores distribution (**b**) and (**d**) for the 200 K marker panel scenario. Horizontal dashed lines indicate the 99.5 (red), 99.0 (blue), and 97.5 (green) quantiles of the F_ST_ distribution

Figure 1 presents the distribution and effects of the 100 QTL simulated from a gamma distribution with a shape parameter of 0.4 (Fig 1a) and the F_ST_ scores for the 200 K SNPs (Fig. 1b). The largest QTL explained about 13.2% of the total genetic variance (GV). The top 15 QTL explained over 70% of the GV while the bottom 50% of QTL explained less than 0.05% of the GV each. The distribution of estimated F_ST_ scores (Fig. 1b) showed a striking similarity to the true QTL distribution (Fig. 1a), especially for large effect QTL. For QTL with effect greater than 0.2 (Fig. 1a) there were three distinguished peaks that are easily captured by the F_ST_ scores under the three threshold cutoff values (Fig. 1b). This result was not unexpected given the large simulated effects for the top QTL. Due to selection, SNPs in LD with these QTL will experience quick and substantial change in their minor allele frequencies that will be easily captured by the F_ST_ scores. When the QTL effects were pre-defined (each QTL explains at least 0.5% of GV), the QTL with the largest effect explained 1.5% of GV and the bottom 50% of QTL explained between 0.5 and 1% of the GV each (Fig. 1c and d). Even under this complex genetic model and absence of large effect QTL, SNPs selected based on F_ST_ scores were able to track the majority of QTL with as little as 3% of all SNP in the panel (Fig. 1d). Similar results were observed when a 400 K SNP panel was considered (Additional file 1: Figure S1).

### Accuracy of genomic selection: Population genetics approach

Table 3 presents the accuracy of prediction of both of the true breeding values and the simulated phenotypes in the case where the SNPs were preselected based on F_ST_ scores. Accuracy was calculated based on the correlation between the true parameters (breeding values or phenotypes) and their associated prediction on the validation data set (G4). All results are based on the average of 5 replicates for each simulated data set. Using all SNPs in the 200 K and 400 K panels resulted in genomic accuracy of 0.777 and 0.775, respectively when the QTL effects were generated from a gamma distribution. When the QTL effects were predefined, the corresponding accuracies were 0.741 and 0.735. This drop in accuracy is due in part to the increased complexity of the genetic model in the case of predefined QTL effects which resulted in a reduction in the portion of GV explained compared to the scenario when QTL were simulated from a gamma distribution. When SNPs were preselected based on their F_ST_ scores under the 200 K marker panel and gamma distribution for the simulation of the QTL effects scenario, genomic accuracy increased from 0.725 to 0.853 when the preselected SNPs were based either on the 99.5 (1076 SNPs) or 97.5 quantile (4932 SNPs) of the distribution of the F_ST_ scores. Similarly, the number of tagged QTL (r^2^ > 0.7 with at least one selected SNP) and the portion of GV explained increased from 13 to 33 and 64.08 to 83.70%, respectively. When the QTL effects were pre-defined to explain at least 0.5% of the GV, the same trend was observed as when the QTL were simulated from a gamma distribution, except that the accuracies and portion of genetic variance explained were smaller and the number of tagged QTL was larger for the same quantile. At the 97.5 quantile, 69% of the QTL were tagged for the predefined scenario versus 33% in the gamma distribution scenario. However, the predefined scenario explained only 71.27% of the GV compared to 83.70% in the gamma distribution scenario. This is obviously due to the change in the complexity of the genetic model. Using the 400 K marker panel, accuracies, number of tagged QTL and portion of GV explained increased compared to the 200 K SNP scenario (Table 3). This is likely due to an increase in LD between preselected SNPs and QTL. However, the difference between the two marker density scenarios is small for the 97.5 quantile case. This indicates that in this case, around 5000 SNPs are needed to track the majority of the QTL and any additional markers will increase accuracy marginally. Across all simulation scenarios, phenotype prediction accuracy has the same trend as the accuracy of genomic enhanced breeding values (GEBV) although with a much lower magnitude, as expected (Table 3). It is worth mentioning that although the optimum number of preselected SNPs was not determined in this study, a continuous increase in the number of markers in the association model will at some point lead to a decrease in accuracy. This is well supported by the lower accuracy when all SNPs were included in the model (Table 3).

**Table 3:** Number of selected SNPs, number of tagged QTLs, percentage of genetic variance explained, and accuracies of genomic and phenotype prediction under different quantile of the distribution of FST scores, sampling distribution for the QTL effects and density of the marker panel using the proposed method. Standard errors of accuracies are listed between parentheses

	All SNPs		97.5 quantile^1^		99.0 quantile		99.5 quantile	
	Gamma^2^	Predefined^3^	Gamma	Predefined	Gamma	Predefined	Gamma	Predefined
200 K SNP marker panel								
Selected SNP	200 K	200 K	4932	5620	1956	2171	935	1076
Tagged QTL^4^	95	97	33	69	18	47	13	31
% GV^5^	91.29	98.60	83.70	71.27	73.57	49.69	64.08	35.10
Acc_P^6^	0.462	0.445	0.503	0.490	0.472	0.415	0.434	0.359
	(0.018)	(0.012)	(0.017)	(0.014)	(0.015)	(0.018)	(0.028)	(0.032)
Acc_G^7^	0.777	0.741	0.853	0.830	0.797	0.704	0.725	0.617
	(0.017)	(0.012)	(0.019)	(0.023)	(0.017)	(0.031)	(0.037)	(0.026)
400 K SNP marker panel								
Selected SNP	400 K	400 K	10,173	10,651	3586	4646	2078	2037
Tagged QTL	95	99	38	74	20	53	13	34
% GV	96.73	99.01	84.03	75.09	73.83	56.66	66.12	43.79
Acc_P	0.456	0.438	0.506	0.485	0.473	0.433	0.448	0.350
	(0.015)	(0.017)	(0.014)	(0.017)	(0.029)	(0.021)	(0.039)	(0.028)
Acc_G	0.775	0.735	0.860	0.813	0.807	0.722	0.765	0.685
	(0.020)	(0.012)	(0.015)	(0.012)	(0.041)	(0.025)	(0.059)	(0.052)

^1^quantile of the distribution of the F_ST_ scores, ^2^QTL effects sampled from a Gamma distribution, ^3^QTL effects pre-defined to explain at least 0.5% of genetic variance (GV), ^4^QTL with r^2^ > 0.7 with at least one selected SNP, ^5^GV = Genetic Variance, ^6^accuracy of phenotype prediction, ^7^accuracy of genomic prediction

**Fig. 2:**
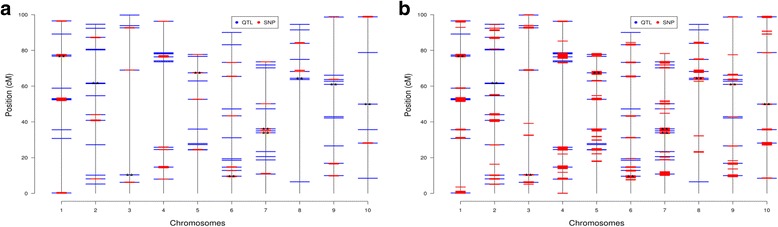
Distribution of the simulated QTL (in Blue) and the preselected SNPs (in Red) across the 10 chromosomes using the 99.5 (**a**) and 97.5 (**b**) quantiles of the F_ST_ scores under the predefined QTL effect and the 200 K marker panel simulation scenario. (* indicates the top 10% QTL)

Figure 2 presents the distribution of simulated QTL across the 10 chromosomes and the preselected SNPs based on 99.5 (Fig. 2a) and 97.5 quantile (Fig. 2b) of the F_ST_ score distribution for the 200 K marker panel and predefined QTL effect scenario (Additional file 2: Figure S2.1, Additional file 3: Figure S2.2, Additional file 4: Figure S2.3 present the results for the remaining scenarios). It is clear that when only SNPs with a large F_ST_ score were preselected (Fig. 2a), only large QTL were tagged. As more SNPs are preselected (Fig. 2b), most of the QTL (70%) were tagged and a large proportion of the GV was explained. When the QTL were simulated from a gamma distribution, although only the most influential QTL were effectively tagged, the majority of the GV was explained even when SNPs were preselected based on their F_ST_ score exceeding the 99.5 quantile of the distribution (Table 3).

In order to further evaluate the performance of the SNP prioritization approach based on F_ST_ scores, a comparison with well-established and extensively used methods was carried out. The same simulated data sets were analyzed using BayesB, and BayesC implemented by GenSel software [34]. For BayesB and BayesC, four π values (0.01, 0.02, 0.05 and 0.10) were evaluated. Table 4 presents the accuracies using BayesB. For both marker densities, the accuracies increased with the decrease of π with the maximum at π = 0.01. For the 200 K SNP scenario, accuracy of predicted GEBV ranged from 0.797 to 0.845 and from 0.770 to 0.833 when QTL effects were simulated from a gamma distribution or predefined, respectively. A similar trend was observed for the 400 K SNP scenario, although the magnitude of the accuracies was slightly smaller. Using BayesC, the results were very similar to those obtained using BayesB, although they tended to be slightly higher for the latter (Table 5). When compared with the proposed method, BayesB and BayesC have slightly lower accuracies of genomic prediction in all simulation scenarios, except the 200 K SNP marker density and predefined QTL effect scenario using BayesB (Table 6). In fact, the superiority in the remaining scenarios ranged from 0.74 to 3.60% and 1.08 to 4.19% compared to BayesB and BayesC, respectively. Similar trend was observed for the phenotype prediction accuracy. Phenotypic accuracy was lower using the proposed method only for the 200 K SNP marker panel and predefined QTL effect scenario (Table 6).

**Table 4:** Number of selected SNPs, number of tagged QTL, percentage of genetic variance explained, and accuracies of genomic and phenotype prediction under different π values, sampling distribution for the QTL effects and density of the marker panel using BayesB method. Standard errors of accuracies are listed between parentheses

	(1-π) =0.90		(1-π) =0.95		(1-π) =0.98		(1-π) =0.99	
	Gamma^1^	Predefined^2^	Gamma	Predefined	Gamma	Predefined	Gamma	Predefined
200 K marker density								
# SNP	20 K	20 K	10 K	10 K	4 K	4 K	2 K	2 K
Tagged QTL^3^	78	98	63	97	54	94	48	91
% GV^4^	89.31	98.16	86.43	97.88	84.30	95.76	83.88	93.20
Acc_P^5^	0.473	0.463	0.478	0.471	0.489	0.487	0.499	0.500
	(0.018)	(0.009)	(0.018)	(0.009)	(0.018)	(0.008)	(0.018)	(0.007)
Acc_G^6^	0.797	0.770	0.807	0.785	0.827	0.810	0.845	0.833
	(0.017)	(0.008)	(0.017)	(0.007)	(0.018)	(0.007)	(0.018)	(0.005)
400 K marker density								
# SNP	40K	40K	20 K	20 K	8 K	8 K	4 K	4 K
Tagged QTL	86	99	75	98	59	97	53	96
% GV	92.36	98.46	91.88	98.16	91.20	97.78	91.03	96.69
Acc_P	0.465	0.450	0.470	0.457	0.478	0.469	0.488	0.481
	(0.015)	(0.018)	(0.015)	(0.018)	(0.014)	(0.018)	(0.013)	(0.019)
Acc_G	0.790	0.756	0.799	0.767	0.813	0.787	0.829	0.807
	(0.019)	(0.013)	(0.017)	(0.013)	(0.016)	(0.014)	(0.015)	(0.014)

^1^ QTL effects sampled from a Gamma distribution, ^2^QTL effects pre-defined to explain at least 0.5% of genetic variance (GV), ^3^QTL with r^2^ > 0.7 with at least one selected SNP, ^4^ GV = Genetic Variance, ^5^ accuracy of phenotype prediction, ^6^accuracy of genomic prediction

**Table 5:** Number of selected SNPs, number of tagged QTL, percentage of genetic variance explained, and accuracies of genomic and phenotype prediction under different π values, sampling distribution for the QTL effects and density of the marker panel using BayesC method. Standard errors of accuracies are listed between parentheses

	(1-π) =0.90		(1-π) =0.95		(1-π) =0.98		(1-π) =0.99	
	Gamma	Predefined	Gamma	Predefined	Gamma	Predefined	Gamma	Predefined
200 K marker density								
# SNP	20 K	20 K	10 K	10 K	4 K	4 K	2 K	2 K
Tagged QTL^3^	76	97	61	96	53	94	46	91
% GV^4^	88.84	97.66	86.56	97.53	86.30	95.74	85.76	93.32
Acc_P^5^	0.453	0.451	0.467	0.459	0.484	0.477	0.496	0.493
	(0.019)	(0.009)	(0.019)	(0.009)	(0.018)	(0.008)	(0.018)	(0.008)
Acc_G^6^	0.769	0.751	0.791	0.766	0.821	0.794	0.842	0.821
	(0.017)	(0.009)	(0.018)	(0.008)	(0.018)	(0.009)	(0.018)	(0.006)
	400 K marker density							
# SNP	40K	40K	20 K	20 K	8 K	8 K	4 K	4 K
Tagged QTL	85	99	68	98	53	97	48	95
% GV	92.05	98.97	91.59	98.37	90.98	96.95	90.16	95.81
Acc_P	0.444	0.441	0.456	0.447	0.472	0.459	0.485	0.472
	(0.013)	(0.017)	(0.013)	(0.017)	(0.014)	(0.017)	(0.014)	(0.018)
Acc_G	0.754	0.740	0.773	0.749	0.802	0.769	0.824	0.791
	(0.017)	(0.011)	(0.017)	(0.011)	(0.017)	(0.012)	(0.016)	(0.012)

^1^QTL effects sampled from a Gamma distribution, ^2^QTL effects pre-defined to explain at least 0.5% of genetic variance (GV), ^3^QTL with r^2^ > 0.7 with at least one selected SNP, ^4^GV = Genetic Variance, ^5^accuracy of phenotype prediction, ^6^accuracy of genomic prediction

**Table 6:** Comparison of best accuracies between BayesB, BayesC, and the proposed method under different sampling distribution for the QTL effects and density of the marker panel

	200 K marker panel		400 K marker panel	
	Gamma^1^	Predefined^2^	Gamma	Predefined
Diff_acc_G^3^				
*BayesB*	−0.94	0.36	−3.60	−0.74
*BayesC*	−1.29	−1.08	−4.19	−2.71
Diff_acc_P^4^				
*BayesB*	−0.80	2.04	−3.56	−0.82
*BayesC*	−1.39	0.61	−4.15	−2.68

^1^QTL effects sampled from a Gamma distribution, ^2^QTL effects pre-defined to explain at least 0.5% of genetic variance, ^3^percentage difference in genomic accuracy compared to the proposed method, ^4^percentage difference in phenotype prediction genomic accuracy compared to the proposed method

In the 200 K SNP marker panel simulation scenario we tried to mimic the LD observed in the Bovine 770 K chip. Thus, simulations were carried out with LD between adjacent SNPs ranging between 0.65–0.70. In order to test the performance of the proposed method when LD is lower, the 200 K SNP marker density and gamma QTL effect scenario was re-simulated with LD between adjacent markers of around 0.3. The results showed that across all three methods (BayesB, BayesC and our proposed method), accuracy decreased by 18 to 20% compared with the scenario with higher LD. Furthermore, the three methods have similar results with a slight superiority (0.53%) for BayesB.

Bayesian methods for prioritizing SNPs rely on sound statistical foundation. However, their performance is expected to decay with the increase in the density of the marker panel at least for two reasons: 1) an increase in the number of unknowns in the association model leading to an increase in the statistical cost of finding the relevant SNPs (non-zero effect SNPs) and 2) an increase in the number of markers in the panel increasing the number of SNPs that are in high LD with the QTL. Consequently, the effect of each QTL will be partitioned across an increasing number of markers leading to smaller effects of associated SNPs. Because these methods rely on the magnitude of the estimated marker effects to determine the relevant SNPs, their performance will undoubtedly decay due to a lack of statistical power. However, the proposed method pre-selects markers based on the change of their minor allele frequencies rather than the magnitude of their effects. Thus, it does not suffer from the problem indicated before, but it is prone to some redundancy in the selected SNPs because markers with very high LD will have similar F_ST_ score.

In this study, a homogeneous population was assumed. The proposed method could be modified in presence of admixed populations. Specifically, in the presence of an admixed population the change in the minor allele frequency (MAF) of SNPs and consequently of Fst scores could be the result of selection pressure on linked QTLs or simply due to difference in MAF between components (breeds) of the population. The latter will not be useful to prioritize SNPs. However, in presence of admixed population we suggest performing within breed SNP prioritization which will take care largely of the difference in MAF. SNPs prioritized in more than one breed (at least those with the largest Fst scores) should be tested for LD phase consistency. This could be a manageable task given the limited number of prioritized SNPs. Furthermore, selected SNPs will have effect only in the subpopulations (breeds) where they were prioritized increasing potentially the power of the association model. However, within subpopulation SNP prioritization could be problematic for breeds with small number of genotyped individuals. In such case grouping for genetically closer breeds could be used for SNP prioritization.

Across all simulation scenarios, we tried to mimic high density SNP panels used in livestock applications where causal variants were assumed not to be genotyped. However, with the recent availability of sequence data large portion of causative variants will be genotyped. Furthermore, these variants could have rare frequencies (MAF <1%). These two issues could have impact on the performance of our method as well as other approaches. However, it is intuitive to think that the Fst method will perform even better because causative variants or those in very high or complete LD with them will, in general, see their minor allele frequencies change more significantly than other variants resulting in higher Fst score and easy prioritization. This might not be the case for competing methods (BayesB and BayesC) were prioritization is based on the effect of variants.

## Conclusion

A continuous increase in the density of SNP marker panels and the availability of whole genome sequence data provide an unprecedented opportunity to dissect the genetic basis of complex traits and to enhance the estimation of genetic merit in animal and plant applications. Unfortunately, this dramatic increase in the available genomic data has created some implementation problems and most importantly did not lead to any significant increase of accuracy of genomic selection using single- and multiple-step approaches. For the latter, the massive increase in the number of explanatory variables has led to an over-parametrization of the association model which resulted in increased co-linearity and loss of statistical power. Together these factors led to no increase in accuracy of genomic selection. Limitations of current models stem from the lack of information on the genotyped individual to prioritize SNPs marker to be considered in the association model. Furthermore, methods based on statistical criteria to filter SNPs will see their performance decay as the marker density increases due to the reduction in the effects of SNPs associated with QTL. Using external information (i.e. gene expression data) is attractive and could compensate for the limited information in the data. Unfortunately, such external information is not always available, often is tissue or time specific, and could have high noise-to-signal ratio. In this study, we proposed using F_ST_ score as an alternative to existing method to prioritize SNPs in high-density marker panels. Although this information is internal to the data, the results of this study suggest that it could provide a reliable tool for prioritization of SNPs.

## Additional files

Additional file 1: Figure S1. Distribution of the simulated quantitative trait loci (QTL) along the ten chromosomes when their effects were simulated from a gamma distribution (a) or predefined (c) and their associated F_ST_ scores distribution (b) and (d) for the 400 K marker panel scenario. Horizontal dashed lines indicate the 99.5 (red), 99.0 (blue), and 97.5 (green) quantiles of the F_ST_ distribution (PDF 265 kb)

Additional file 2: Figure S2. 1 Distribution of the simulated QTL (in Blue) and the preselected SNPs (in Red) across the 10 chromosomes using the 99.5 (a) and 97.5 (b) quantiles of the F_ST_ scores under the QTL effect sampled a Gamma distribution with shape parameter equal to 0.4. and the 200 K marker panel simulation scenario. (* indicates the top 10% QTL) (PDF 196 kb)

Additional file 3: Figure S2. 2 Distribution of the simulated QTL (in Blue) and the preselected SNPs (in Red) across the 10 chromosomes using the 99.5 (a) and 97.5 (b) quantiles of the F_ST_ scores under the QTL effect sampled a Gamma distribution with shape parameter equal to 0.4. and the 400 K marker panel simulation scenario. (* indicates the top 10% QTL) (PDF 201 kb)

Additional file 4: Figure S2. 3 Distribution of the simulated QTL (in Blue) and the preselected SNPs (in Red) across the 10 chromosomes using the 99.5 (a) and 97.5 (b) quantiles of the F_ST_ scores under the predefined QTL effect and the 400 K marker panel simulation scenario. (* indicates the top 10% QTL) (PDF 202 kb)

## Abbreviations

EBVs: Estimated breeding values
GEBV: Genomic enhanced breeding values
GS: Genomic selection
GV: Genetic variance
GWAS: Genome-wide association study
HD: High-density
LD: Linkage disequilibrium
NGS: Next generation sequencing
QTL: Quantitative trait locus
RM: Multiple regression
SNPs: Single nucleotide polymorphisms
VC: Variance component
