# Correction to: High density marker panels, SNPs prioritizing and accuracy of genomic selection

**DOI:** 10.1186/s12863-018-0598-7

**Published:** 2018-02-15

**Authors:** Ling-Yun Chang, Sajjad Toghiani, Ashley Ling, Sammy E. Aggrey, Romdhane Rekaya

**Affiliations:** 1 Department of Animal and Dairy Science, University of Georgia, Athens, GA 30602 USA; 2 Department of Poultry Science, University of Georgia, Athens, GA 30602 USA; 3 Institute of Bioinformatics, University of Georgia, Athens, GA 30602 USA

## Correction to: BMC Genetics (2018) 19:4 DOI: 10.1186/s12863-017-0595-2

The original version of this article [1], published on 5 January 2018, contained 3 formatting errors. In this Correction the affected parts of the article are shown. The original article has been updated.

Table 1 contained a formatting error. The correct version of Table 1 is shown below and the corrected entry is marked in bold:

**Table 1:** Descriptive statistics of simulation schemes

Historical Population (HP)	
Number of generation	315
Mutation rate for markers	1.0*10^− 4^
Mutation rate for QTL	1.0*10^**−4**^
Founder Population (G0)	
Number of generation	3
Number of male	1500
Number of female	15,000
Selection Population (G3)	
Number of chromosomes	10
Length per chromosome (cM)	100
Number of markers per generation	200,000 / 400,000
Marker distribution	Evenly spaced
Number of QTL per generation	100
QTL distribution	Randomly distributed
QTL effect	Sampled from gamma with shape 0.4
Heritability	0.4
Genetic variance	0.4
Residual variance	0.6

0

Table 5 contained a formatting error. The correct version of Table 5 is shown below and the corrected entry is marked in bold:

**Table 5:** Number of selected SNPs, number of tagged QTL, percentage of genetic variance explained, and accuracies of genomic and phenotype prediction under different π values, sampling distribution for the QTL effects and density of the marker panel using BayesC method. Standard errors of accuracies are listed between parentheses

	(1-π) =0.90		(1-π) =0.95		(1-π) =0.98		(1-π) =0.99	
	Gamma	Predefined	Gamma	Predefined	Gamma	Predefined	Gamma	Predefined
200 K marker density								
# SNP	20 K	20 K	10 K	10 K	4 K	4 K	2 K	2 K
Tagged QTL^3^	76	97	61	96	53	94	46	91
% GV^4^	88.84	97.66	86.56	97.53	86.30	95.74	85.76	93.32
Acc_P^5^	0.453	0.451	0.467	0.459	0.484	0.477	0.496	0.493
	(0.019)	(0.009)	(0.019)	(0.009)	(0.018)	(0.008)	(0.018)	(0.008)
Acc_G^6^	0.769	0.751	0.791	0.766	0.821	0.794	0.842	0.821
	(0.017)	(0.009)	(0.018)	(0.008)	(0.018)	(0.009)	(0.018)	(0.006)
**400 K marker density**								
# SNP	40K	40K	20 K	20 K	8 K	8 K	4 K	4 K
Tagged QTL	85	99	68	98	53	97	48	95
% GV	92.05	98.97	91.59	98.37	90.98	96.95	90.16	95.81
Acc_P	0.444	0.441	0.456	0.447	0.472	0.459	0.485	0.472
	(0.013)	(0.017)	(0.013)	(0.017)	(0.014)	(0.017)	(0.014)	(0.018)
Acc_G	0.754	0.740	0.773	0.749	0.802	0.769	0.824	0.791
	(0.017)	(0.011)	(0.017)	(0.011)	(0.017)	(0.012)	(0.016)	(0.012)

^1^QTL effects sampled from a Gamma distribution, ^2^ QTL effects pre-defined to explain at least 0.5% of genetic variance (GV), ^3^ QTL with r^2^ > 0.7 with at least one selected SNP, ^4^ GV = Genetic Variance, ^5^ accuracy of phenotype prediction, ^6^ accuracy of genomic prediction

The first equation contained formatting errors. The correct version of this equation is shown below:

$$ {F}_{ST}=\frac{H_T-{H}_S}{H_T} $$

with *H*_*T*_ = 2 ∗ *p* ∗ *q*, ${H}_S=\frac{H_{S1}\ast {n}_{S1}+{H}_{S2}\ast {n}_{S2}}{n_{S1}+{n}_{S2}}$, and *H*_*Si*_ = 2 ∗ *p*_*Si*_ ∗ *q*_*Si*_
