# Supplementary figures and images for: High density marker panels, SNPs prioritizing and accuracy of genomic selection
Source: BMC Genet. 2018 Jan 5;19:4. doi: 10.1186/s12863-017-0595-2 (PMC5756446; doi:10.1186/s12863-017-0595-2)

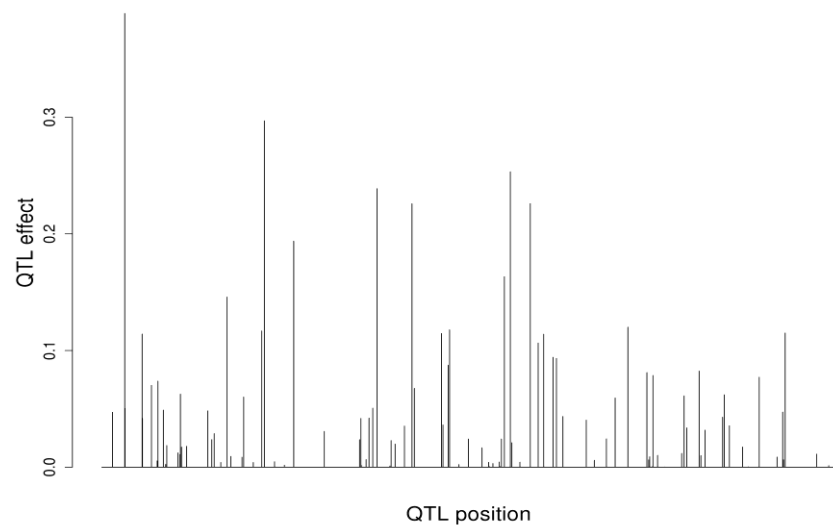

(a)

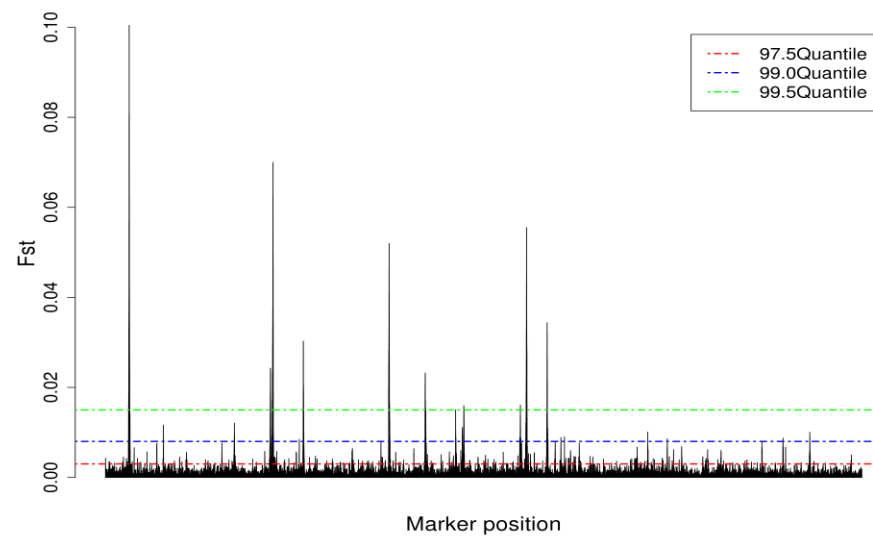

(b)

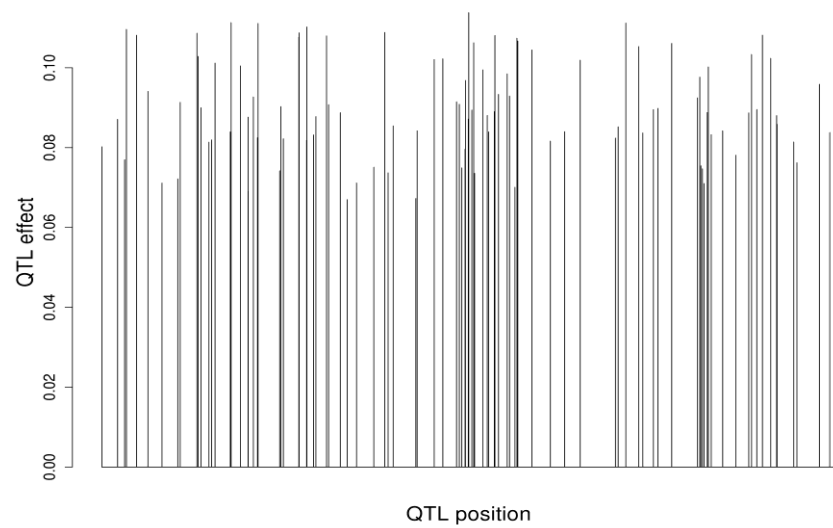

(c)

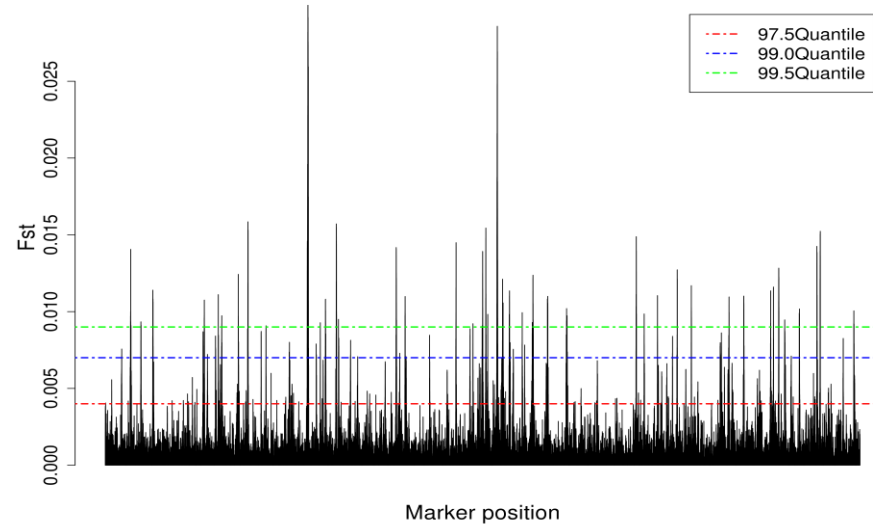

(d)

Supplement: Supplementary file 1 — Distribution of the simulated quantitative trait loci (QTL) along the ten chromosomes when their effects were simulated from a gamma distribution (a) or predefined (c) and their associated FST scores distribution (b) and (d) for the 400 K marker panel scenario. Horizontal dashed lines indicate the 99.5 (red), 99.0 (blue), and 97.5 (green) quantiles of the FST distribution (PDF 265 kb) [file 12863_2017_595_MOESM1_ESM.pdf]

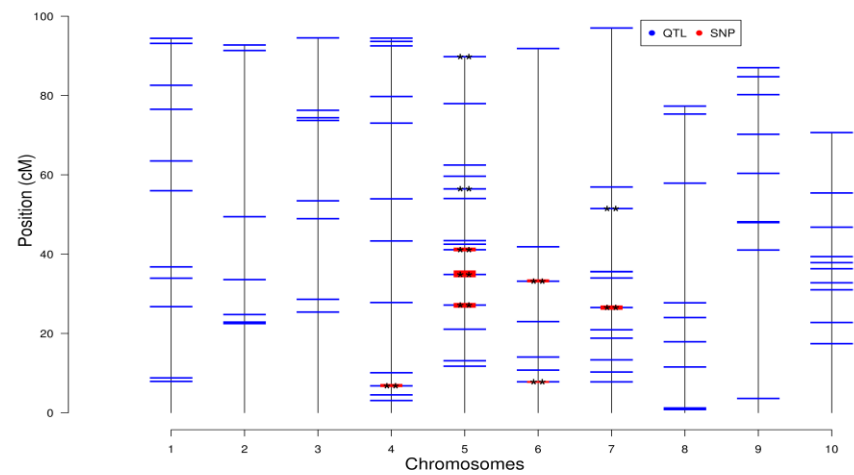

(a)

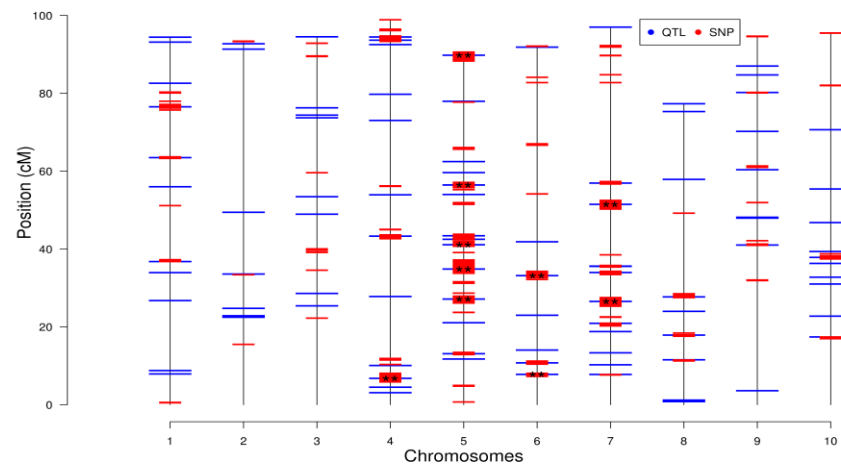

(b)

Supplement: Supplementary file 2 — 1 Distribution of the simulated QTL (in Blue) and the preselected SNPs (in Red) across the 10 chromosomes using the 99.5 (a) and 97.5 (b) quantiles of the FST scores under the QTL effect sampled a Gamma distribution with shape parameter equal to 0.4. and the 200 K marker panel simulation scenario. (* indicates the top 10% QTL) (PDF 196 kb) [file 12863_2017_595_MOESM2_ESM.pdf]

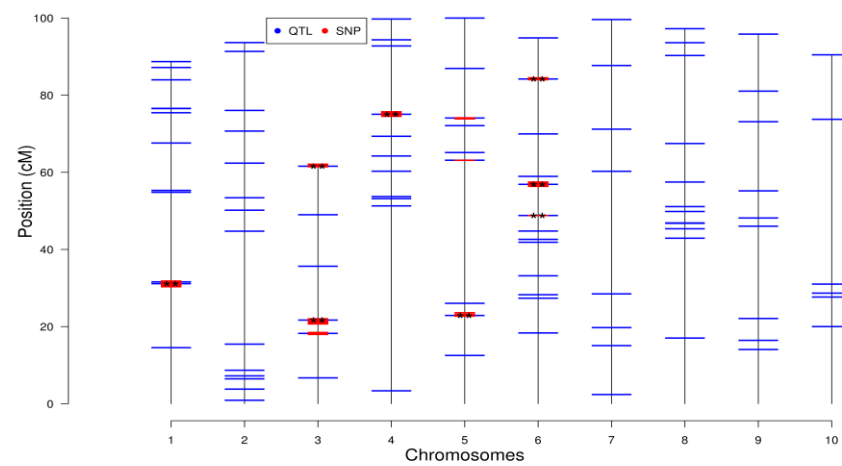

(a)

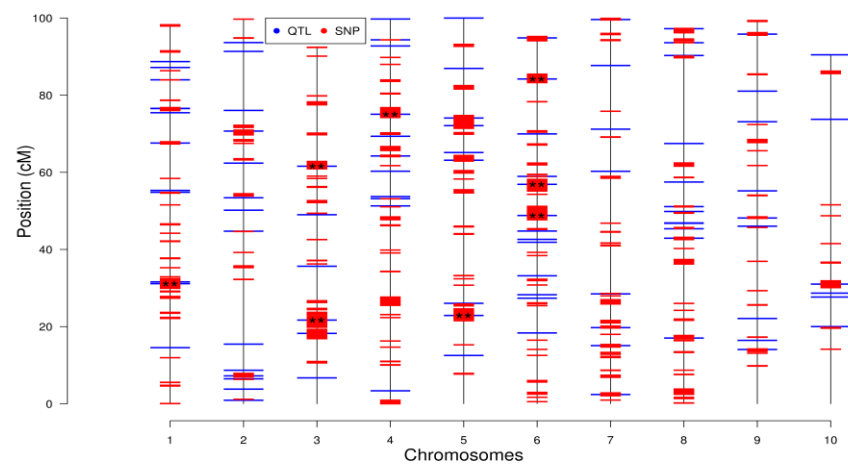

(b)

Supplement: Supplementary file 3 — 2 Distribution of the simulated QTL (in Blue) and the preselected SNPs (in Red) across the 10 chromosomes using the 99.5 (a) and 97.5 (b) quantiles of the FST scores under the QTL effect sampled a Gamma distribution with shape parameter equal to 0.4. and the 400 K marker panel simulation scenario. (* indicates the top 10% QTL) (PDF 201 kb) [file 12863_2017_595_MOESM3_ESM.pdf]

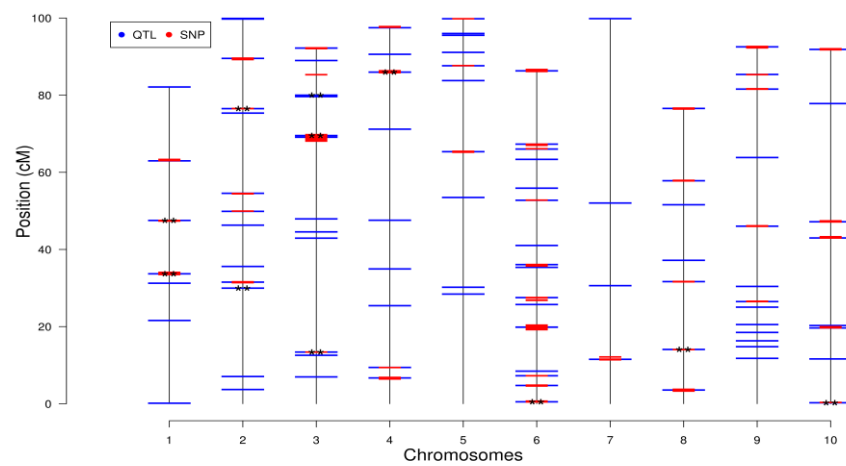

(a)

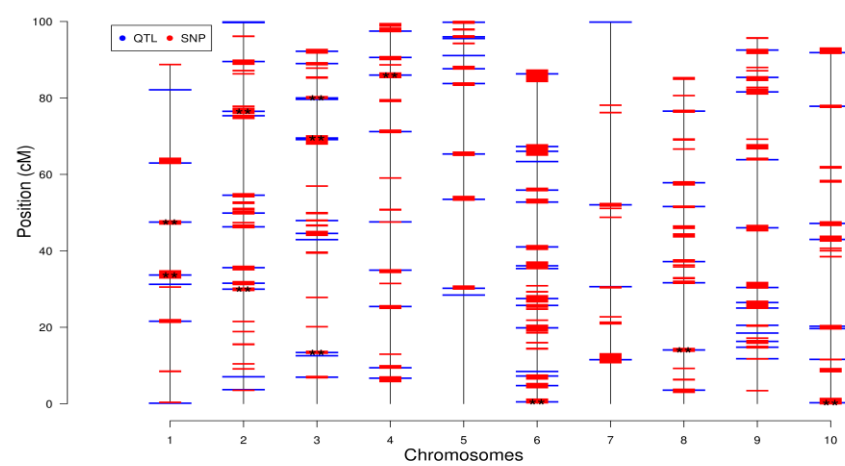

(b)

Supplement: Supplementary file 4 — 3 Distribution of the simulated QTL (in Blue) and the preselected SNPs (in Red) across the 10 chromosomes using the 99.5 (a) and 97.5 (b) quantiles of the FST scores under the predefined QTL effect and the 400 K marker panel simulation scenario. (* indicates the top 10% QTL) (PDF 202 kb) [file 12863_2017_595_MOESM4_ESM.pdf]
